# ARPKD and early manifestations of ADPKD: the original polycystic kidney disease and phenocopies

**DOI:** 10.1007/s00467-013-2706-2

**Published:** 2014-03-01

**Authors:** Carsten Bergmann

**Affiliations:** 1 Center for Human Genetics, Bioscientia, Konrad-Adenauer-Str. 17, 55218 Ingelheim, Germany; 2 Department of Nephrology and Center for Clinical Research, University Hospital Freiburg, Freiburg, Germany

**Keywords:** Polycystic kidney disease (ADPKD/ARPKD), Ciliopathies, HNF1ß, Nephronophthisis, Bardet–Biedl syndrome, Joubert syndrome and related disorders, Next-generation sequencing

## Abstract

Renal cysts are clinically and genetically heterogeneous conditions. Polycystic kidney disease (PKD) is common and its characterization has paved the way for the identification of a growing number of cilia-related disorders (ciliopathies) of which most show cystic kidneys. While the recessive form of PKD (ARPKD) virtually always presents in childhood, early onset can, in some instances, also occur in the dominant form (ADPKD). Both ADPKD genes (*PKD1* and *PKD2*) can also be inherited in a recessive way, making the story more complex with evidence for a dosage-sensitive network. Several phenocopies are known, and mutations in *HNF1ß* or genes that typically cause other ciliopathies, such as nephronophthisis, Bardet–Biedl, Joubert syndrome and related disorders, can mimic PKD. An accurate genetic diagnosis is crucial for genetic counseling, prenatal diagnostics, and the clinical management of patients and their families. The increasing number of genes that have to be considered in patients with cystic kidney disease is challenging to address by conventional techniques and largely benefits from next-generation sequencing-based approaches. The parallel analysis of targeted genes considerably increases the detection rate, allows for better interpretation of identified variants, and avoids genetic misdiagnoses.

## Classification and differential diagnosis of cystic kidneys

In their seminal studies, Osathanondh and Potter systematically classified renal cystic diseases into four distinct types [1]. Potter syndrome type I is referred to as autosomal recessive polycystic kidney disease (ARPKD), type II as renal cystic dysplasia, type III as autosomal dominant polycystic kidney disease (ADPKD), and type IV occurs when a longstanding obstruction in either the kidney or ureter leads to cystic kidneys or hydronephrosis. Particularly types II–IV can be part of many syndromes. While this classification still has an impact for pathoanatomical descriptions, it is hardly to be reconciled with clinical and genetic entities and, consequently, it is more and more being replaced by the genetic nomenclature.

An accurate diagnosis is essential to both the management of patients with cystic kidneys and the counseling of their families. When an effort is made to classify the wide array of different entities with renal cysts, it might be helpful to first distinguish between acquired and inherited forms. Knowledge of the family history and the clinical picture, together with the location and morphology of the cysts, and any possible extrarenal manifestations should help the treating physician in the decision-making process. In patients with additional extrarenal features (in particular those with growth retardation, learning difficulties, and/or microcephaly), cytogenetic or array comparative genomic hybridization (array CGH) studies may be useful to exclude rearrangements or aberrations such as large deletions or duplications.

Inherited cystic kidney disorders mainly include ADPKD and ARPKD, glomerulocystic kidney disease, and entities comprising the medullary cystic kidney disease–nephronophthisis complex. Notably, cystic kidneys are an important feature of numerous genetic syndromes, such as the mainly recessively inherited ciliopathies Bardet–Biedl, Joubert, Meckel, and Jeune syndromes or the dominant disorders of tuberous sclerosis (TSC), von Hippel–Lindau (VHL) disease, and branchio-oto-renal syndrome.

## Polycystic kidney disease

**Fig. 1:**
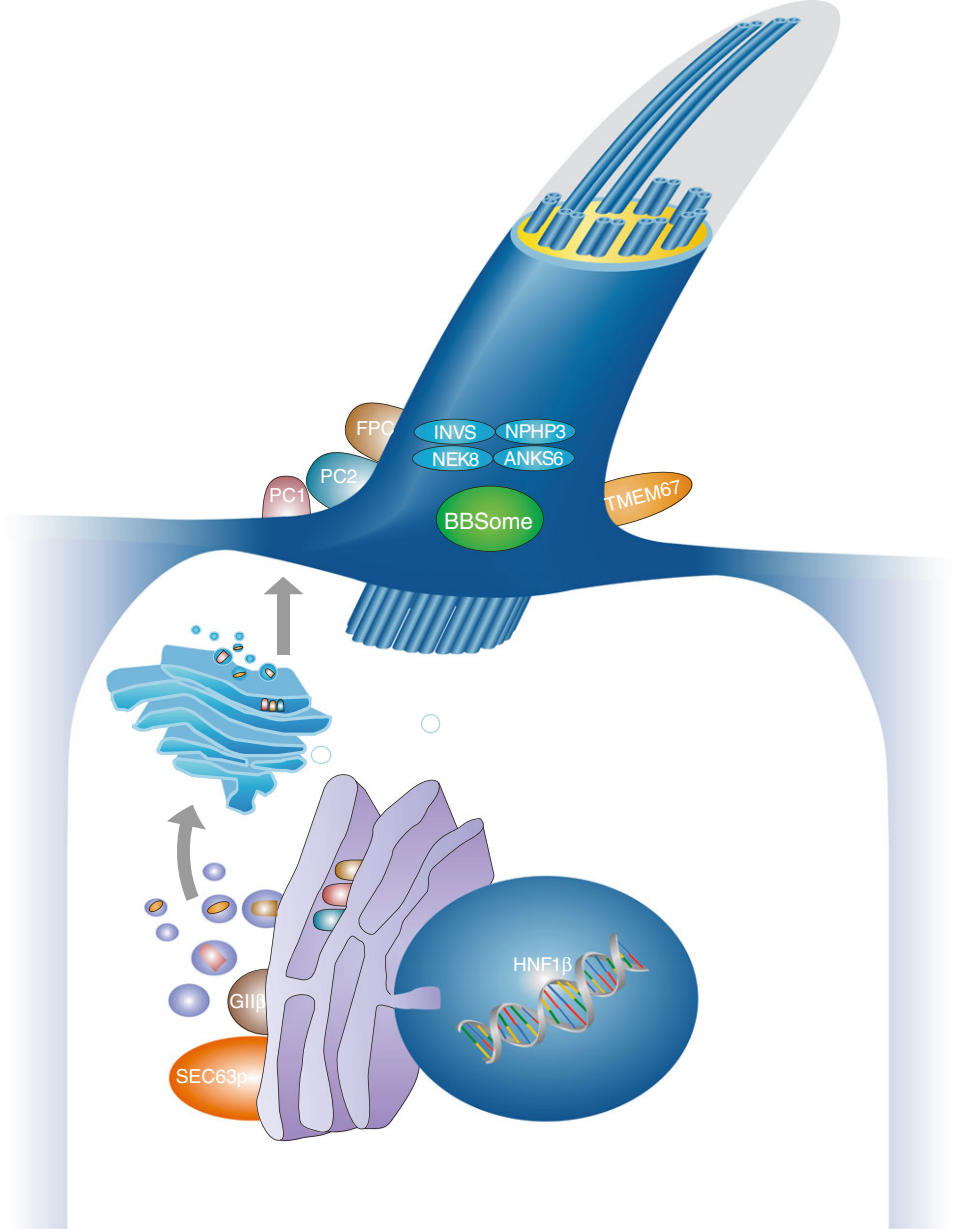
Schematic diagram of a primary cilium and associated processes. Polycystic kidney disease (PKD) is controlled by a defined network of different genes/proteins discussed in this review. Cilia are small antennae that detect a variety of different extracellular stimuli and orchestrate multiple signaling pathways with nuclear trafficking of some molecules [e.g., C-termini of polycystin-1 (*PC1*) and fibrocystin/polyductin (*FPC*)]. The inner ciliary structure is defined by the axoneme that is composed of nine microtubule doublets derived from the basal body and mother centriole of the centrosome. Along this microtubule core, the transport of proteins in the anterograde direction toward the tip of the cilium and in the retrograde direction towards the cell body is organized by an elaborate process called intraflagellar transport

More than 10 years ago, characterization of polycystic kidneys paved the way for the identification of a growing number of cilia-related disorders. It is becoming increasingly evident that most ciliopathies have a renal cystogenic component, making kidney cyst formation a hallmark feature of ciliopathies (Fig. 1) [2, 3]. ADPKD is the most frequent life-threatening genetic disease, with a prevalence of 1/500–1,000 live births, affecting approximately 12.5 million individuals worldwide [4]. About 5–10 % of adult patients who require renal replacement therapy are affected by ADPKD that is usually transmitted in an autosomal dominant fashion. Clinical symptoms typically do not arise until adulthood; however, there is striking phenotypic variability even within the same family, indicating that modifying genes, epigenetic mechanisms, and stochastic and/or environmental factors considerably influence the clinical course in ADPKD. About 2 % of ADPKD patients present with early clinical manifestations before age 15 years [3]. Among these are cases with significant perinatal morbidity and mortality sometimes indistinguishable from that due to severe ARPKD. ARPKD is much rarer than its dominant counterpart and has a suspected incidence of about 1 in 20,000 live births among Caucasians, corresponding to a carrier frequency of approximately 1:70 in non-isolated populations. Isolated and inbred populations may have considerably higher prevalences. For example, Kaariainen and colleagues reported an incidence of 1:8,000 in Finland [5]. In contrast to ADPKD, ARPKD is typically an infantile disease. Notably, among all children with PKD in departments of pediatric nephrology, the total number of patients with ARPKD equals the number of individuals affected with early-onset ADPKD [3].

## Autosomal recessive PKD

**Fig. 2:**
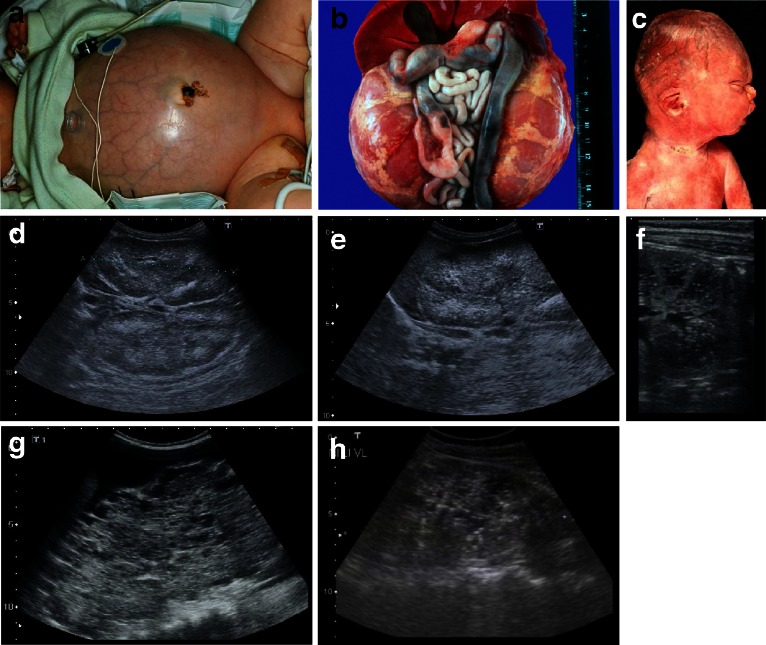
**a**–**h** Autosomal recessive polycystic kidney disease (ARPKD). **a** Baby with distended abdomen due to voluminous kidneys that led to respiratory problems and early demise. **b** Abdominal situs of a perinatally demised ARPKD patient with symmetrically enlarged kidneys that maintained their reniform configuration. **c** Potter’s phenotype with distinctive facial features due to a lack of amniotic fluid. **d**–**h** Renal ultrasound scans of babies and young children with ARPKD. Symmetrically enlarged echogenic kidneys with fusiform dilations of collecting ducts and distal tubules are arranged radially throughout the renal parenchyma from medulla to cortex

Although the clinical spectrum in ARPKD is much more variable than generally presumed, and even only moderately affected elderly people have been described [6, 7], the majority of patients are severely affected and ARPKD is identified late in pregnancy or at birth. Affected fetuses display a “Potter” oligohydramnios phenotype with massively enlarged kidneys, pulmonary hypoplasia, a characteristic facies, and contracted limbs with club feet. Approximately 30–50 % of affected neonates die shortly after birth from respiratory insufficiency due to pulmonary hypoplasia and thoracic compression by the excessively enlarged kidneys (Fig. 2a–c).

**Fig. 3:**
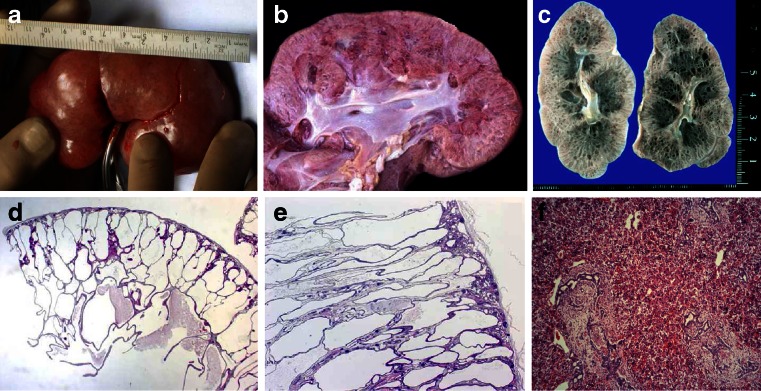
**a**–**f** Microscopic and macroscopic appearance of autosomal recessive polycystic kidney disease (ARPKD). **a** Considerably enlarged kidney with retained reniform configuration (patient passed away at the age  of  2.5 months). **b**, **c** Cross sections of ARPKD kidneys reveal cortical extension of fusiform or cylindrical spaces arranged radially throughout the renal parenchyma from medulla to cortex. **d**, **e** Microscopic appearance of fusiform dilations of renal collecting ducts and distal tubuli lined by columnar or cuboidal epithelium. These dilated collecting ducts run perpendicular to the renal capsule. **f** Obligatory hepatobiliary changes in ARPKD subsumed as ductal plate malformation and characterized by dysgenesis of the hepatic portal triad with hyperplastic biliary ducts and congenital hepatic fibrosis

Ultrasound scans typically show bilaterally enlarged hyperechoic kidneys with poor corticomedullary differentiation, retained reniform contour, and multiple tiny cysts confined to distal tubules and collecting ducts (Fig. 2d–g; Table 1). Macroscopically, the cut surface demonstrates cortical extension of fusiform or cylindrical spaces arranged radially throughout the renal parenchyma from the medulla to the cortex (Fig. 3a, b). Invariable histological manifestations are fusiform dilations of renal collecting ducts and distal tubuli lined by columnar or cuboidal epithelium that usually remain in contact with the urinary system (unlike ADPKD), whereas glomerular cysts (as in ADPKD) or dysplastic elements (e.g., cartilage; as in other ciliopathies such as Meckel syndrome) are usually not evident in ARPKD kidneys (Fig. 3c, d). However, during early fetal development, a transient phase of proximal tubular cyst formation has been identified that is largely absent by birth [8]. With advancing clinical course the kidney structure might increasingly resemble the pattern observed in ADPKD with renal cysts that vary considerably in size and appearance, often also accompanied by some degree of interstitial fibrosis [9].

**Table 1:** Characteristics of autosomal recessive and autosomal dominant polycystic kidney disease

Characteristics	ARPKD	ADPKD
Synonyms	Potter type I	Potter type III
Incidence	Approx. 1:20.000	1:500–1.000 (approx. 2 % early manifesting)
Pathology of kidneys		
Macroscopy	Massively, symmetrically enlarged kidneys (reniform)	Generally enlarged (also reniform), but usually to a lesser extent
Location of cysts	Dilated collecting ducts and distal tubules	Cysts in all parts of the nephron (including glomerulus)
Ultrasound and diameter of cysts	At onset, typical pepper–salt pattern evident on ultrasound scan, increased echogenicity of renal parenchyma throughout cortex and medulla due to tiny, sometimes invisible cysts (usually <2 mm); with advancing age, cysts up to several centimeters large appear, similar to ADPKD pattern	Cysts of different size in cortex and medulla (usually several larger cysts in adults); at onset often small, however, sometimes already several centimeters early in childhood
Pathology of liver	Mandatory: ductal plate malformation/congenital hepatic fibrosis with hyperplastic biliary ducts and portal fibrosis (may impress as Caroli disease)	“Liver cysts” common in adults, but rare in children. Occasionally, ductal plate malformation/congenital hepatic fibrosis
Associated anomalies	Rarely pancreatic cysts and/or fibrosis; single case reports with intracranial aneurysms	Pancreatic cysts and/or cysts in other epithelial organs; intracranial aneurysms in approx. 8 %, familial clustering
Main clinical manifestations	Peri-/neonatal period: respiratory distress (30–50 % of cases)	General onset 3rd–5th decade with arterial hypertension, proteinuria, hematuria, and/or renal insufficiency; approx. 2 % early manifestation in childhood (rarely with perinatal respiratory distress)
	With prolonged survival, renal insufficiency, portal hypertension, and other variable co-morbidities	
Risk for siblings	25 %	50 % (except for rare cases of spontaneous mutation with virtually no risk)
Risk for own children	<1 % (unless unaffected parent is related to his/her affected partner, or ARPKD is known in the unaffected partner’s family)	50 % (also for patients with a spontaneous mutation)
Manifestation in affected family members	Often similar clinical course in siblings (however, in approx. 20 % extensive intrafamilial variability)	Variable, however, often similar within the same family; in the case of early manifestation approx. 50 % recurrence risk
Parental kidneys	No alterations	Except for cases of spontaneous mutation, usually one parent is affected and shows renal cysts (be careful when parents are too young for definite clinical diagnosis, namely, <30–40 years)
Prognosis	In perinatal cases with respiratory distress, usually poor; for those surviving the neonatal period, much better with renal death in approx. 15–30 % in childhood/early adolescence, often severe complications (e. g., esophageal varices) due to portal hypertension; if possible transplantation (often combined kidney–liver TX)	In early manifesting cases, often better than in ARPKD. In “adult” cases, chronic renal failure in approx. 50 % by age of 60 years; median age of ESRD onset (58.1 vs. 79.9 years in PKD1 vs. PKD2)

ARPKD, Autosomal recessive polycystic kidney disease; ADPKD, autosomal dominant polycystic kidney disease; ESRD, end stage renal disease; TX, transplant

Arterial hypertension, often difficult to control despite multi-drug treatment, usually develops during the first months of life and affects up to 80 % of children with ARPKD. Careful blood pressure monitoring is essential to prevent sequelae of hypertension (e.g. cardiac hypertrophy, congestive heart failure), and deterioration of the renal function [7].

While the early appearance of ARPKD is typically clearly dominated by renal manifestations and associated co-morbidities, histological liver involvement is present in every ARPKD patient from early embryonic development on, as reflected by the disease term “polycystic kidney and hepatic disease 1 (PKHD1).” These obligatory liver changes are characterized by defective remodeling of the ductal plate with congenital hepatic fibrosis and biliary duct ectasia [10, 11], defined as ductal plate malformation (DPM) (Fig. 3e) which is also a frequent feature in other ciliopathies such as, for example, Bardet–Biedl, Joubert, Meckel, and Jeune syndrome. Biliary anomalies may develop at any stage of the physiological involution–remodeling process, and the timing or stage of development determines the resulting clinical and histological phenotype [12].

Apart from the portal tracts, the remaining liver parenchyma in ARPKD usually develops normally and liver enzymes, except for cholestasis parameters that might be elevated, are characteristically not increased. The hepatobiliary complications may dominate the clinical picture, particularly in older patients. DPM leads to progressive hepatic fibrosis and consecutive portal hypertension that causes hypersplenism with pancytopenia and esophageal varices with upper gastrointestinal bleeding [7, 13]. Primary management of variceal bleeding includes endoscopic approaches, such as sclerotherapy or variceal banding. Occasionally, portosystemic shunting or liver–kidney transplantation (sequential or combined) has to be considered. ARPKD patients with liver cysts due to extensive dilatations of intra- and extrahepatic bile ducts (Caroli’s disease and syndrome) are at increased risk of recurrent episodes of ascending bacterial cholangitis that may cause fulminant hepatic failure [14].

Although most patients show a comparable degree of severity with regard to liver and kidney affection (renal–hepatobiliary morbidity pattern, such as in a patient with renal insufficiency, it is highly likely that also the liver is considerably affected and vice versa), there is no direct correlation or interdependency between those two organs. Single ARPKD patients may present with an organ-specific phenotype, i.e., either an (almost) exclusive renal phenotype or a predominant or mere liver phenotype. In accordance with these observations, it has been demonstrated that *PKHD1* mutations can cause isolated congenital hepatic fibrosis or Caroli’s disease [7, 15]. It is noteworthy that two transgenic mouse models for *Pkhd1* display an isolated liver phenotype without any renal involvement [16].

To date, *PKHD1* is the only known gene for classical ARPKD, but there is compelling evidence for locus heterogeneity and phenocopies. Thus, single heterozygous mutations and results that are merely based on linkage need to be interpreted with caution. Patients with two truncating mutations generally display a severe phenotype with peri- or neonatal death, whereas patients surviving the neonatal period usually carry at least one hypomorphic (missense) mutation. Due to allelic heterogeneity and a high level of missense mutations and private changes, mutation analysis for *PKHD1* is still laborious, but it has greatly benefited from the availability of new sequencing techniques.

*PKHD1* is a large gene that extends over a genomic segment of almost 500 kb on chromosome 6p12. The longest open reading frame comprises 66 exons that encode polyductin/fibrocystin, a type I single-pass transmembrane protein of 4,074 amino acids [17, 18]. In common with both ADPKD proteins (polycystin-1 and polycystin-2) and most other cystoproteins, fibrocystin is localized to primary cilia with the highest concentration in the basal body area [19]. This striking pattern of subcellular localization and known interactions with, for example, polycystin-2, place fibrocystin at key sites of microtubule organization. In line with its proposed role as a ciliary-localized membrane protein, an 18-residue motif in the cytoplasmic tail of fibrocystin serves as a ciliary targeting signal [20].

## Autosomal dominant PKD

The majority of ADPKD patients (approx. 80–85 %) carry a germline mutation in the *PKD1* gene on chromosome 16p13, whereas about 15–20 % harbor a mutation in the *PKD2* gene on chromosome 4q21 [4]. As in *PKHD1*, there is also a high level of allelic heterogeneity in both ADPKD genes with most mutations being private. About 70 % of all *PKD1* mutations and even more than 80 % of all *PKD2* mutations currently listed in the ADPKD mutation database are predicted to be of a protein truncating character.

The encoded proteins polycystin-1 and polycystin-2 are both glycosylated integral membrane proteins that interact via their C-terminal coiled–coil domains. Polycystin-2 is a member of the transient receptor potential (TRP) protein superfamily and known to function as a divalent cation channel that is particularly involved in cellular Ca^2+^ signaling [21]. While the definite roles of polycystin-1 and the ARPKD protein fibrocystin are still speculative, there is increasing evidence from mutational and functional data that fibrocystin is part of the polycystin complex [22–25].

*PKD1* sequencing is complicated by the presence of six pseudogenes in a duplicated region adjacent to the original *PKD1* locus. It is still a matter of debate if these pseudogenes are merely “junk” or functional DNA. Several lines of evidence show that some pseudogenes are “alive” with functional roles in gene expression and regulation, such as by acting as microRNA decoys [26]. Mutation analysis in ADPKD has much improved during recent years, and it is now generally possible to detect the disease-causing mutation in most affected families. Sequencing of the large and structurally complex *PKD1* gene is usually the first step; if negative, it is followed by *PKD2* sequencing and finally MLPA (multiplex ligation-dependent probe amplification) analysis of both genes to detect larger deletions.

While clinical symptoms usually only arise in adulthood, there is considerable phenotypic variability even within the same family, and in about 2 % of ADPKD patients the symptoms become manifest during childhood. Notably, affected families with early-manifesting offspring have a high recurrence risk for the birth of a another child with similar clinical manifestations. This important piece of information should be shared with afflicted families and clearly hints at a common familial modifying background for early and severe disease expression [27]. Early disease manifestation in ADPKD is the most important differential diagnosis of ARPKD. Parental renal ultrasound is a must and should be performed in every child with cystic kidney disease of unknown origin. However, the family history does not need to be positive because some alleles are incompletely penetrant (hypomorphic), and approximately 2–5 % of all mutations in ADPKD are thought to arise de novo. Moreover, both ADPKD genes can also be inherited in a recessive way [3].

**Fig. 4:**
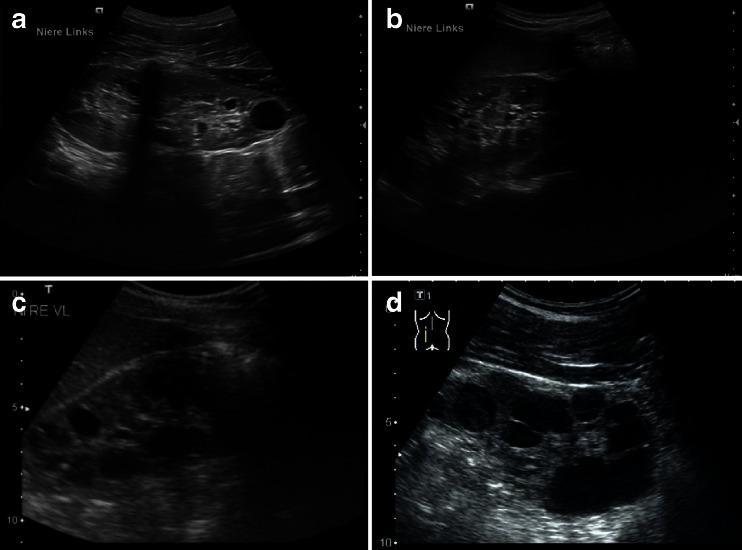
Typical sonographic images of autosomal dominant polycystic kidney disease (ADPKD) in different patients of adolescent age

**Fig. 5:**
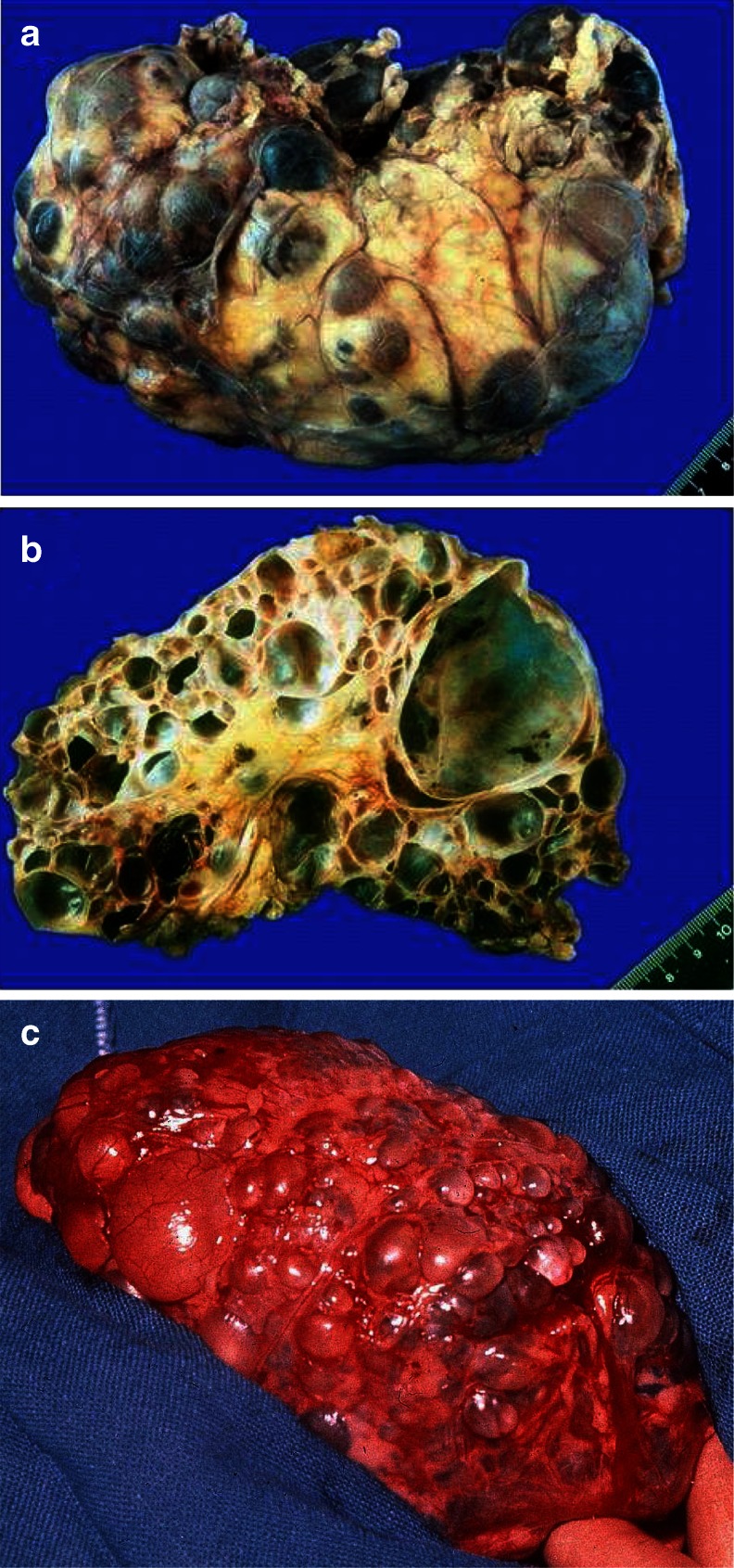
**a**, **c** Macroscopic appearance of advanced-stage autosomal dominant polycystic kidney disease (ADPKD) showing enlarged kidneys with multiple cysts that almost completely destroyed and replaced the renal parenchyma. **b** On cut section, multiple cysts in the cortex and medulla can be seen that vary considerably in size and appearance, from a few millimeters to diameters of many centimeters

Diagnosis of ADPKD by ultrasound is established in at-risk individuals aged 15–39 years if three or more (unilateral or bilateral) renal cysts are detected. About 60 % of children aged <5 years, and 75–80 % of children aged 5–18 years with a known *PKD1* mutation had renal cysts detectable by ultrasound. In general, the finding of even one renal cyst should alert a pediatrician to the possibility of ADPKD because simple cysts are otherwise extremely rare in childhood. In children with a 50 % risk of ADPKD, the finding of one cyst can thus be considered diagnostic [28]. Renal cysts can vary considerably in size and appearance arising from all segments of the nephron (Figs. 4, 5), and they usually show progressive enlargement and may become disconnected from the tubular space (Fig. 5). The Consortium for Radiologic Imaging for the Study of Polycystic Kidney Disease (CRISP) recently showed that renal enlargement in ADPKD mimicked exponential growth. Over a 3-year period among more than 200 patients, the renal volume increased by a mean of 63.4 ml (5.3 %) per year as a result of the expansion of cysts in a continuous process associated with the decline of renal function. While this leads to progressive destruction of the adjacent renal parenchyma and massive enlargement of the kidneys, renal function can be preserved for a long time as functioning nephrons undergo compensatory hypertrophy [29]. Chronic renal failure presents in about 50 % of patients by the age of 60 years. PKD2 is usually significantly milder than PKD1, with the development of end-stage renal disease (ESRD) at a later age and a lower prevalence of arterial hypertension and urinary tract infections. Patients with PKD1 develop ESRD approximately 20 years earlier than those with PKD2, with a median age of onset of ESRD of 58.1 years and 79.9 years, respectively. The greater severity of PKD1 is due to the development of more cysts at an early age—not to faster cyst growth [30]. A recent study on more than 500 pedigrees with ADPKD has provided important information and demonstrated that the type of *PKD1* mutation significantly correlates with renal survival [31]. In that study, patients with a non-truncating mutation exhibited ESRD 12 years later than those with a truncating mutation in the same gene (67.9 vs. 55.6 years).

While the kidneys are the main organ involved, ADPKD is clearly a systemic disorder with profound extrarenal disease burden. Polycystic liver disease is by far the most common extrarenal manifestation in ADPKD and is usually a benign disease that may cause mechanical compression or irritation. Notably, polycystic liver disease can also occur in isolation as an autosomal dominant trait with a mutation in either *PRKCSH* or *SEC63* encoding non-ciliary proteins with roles in protein translocation, folding, and quality control in the endoplasmic reticulum. All PKD genes are transmembrane proteins passing through the translocation and quality control machinery in the ER. Thus, PKD and PLD overlap not only clinically but also genetically and functionally, with evidence for a dosage-sensitive network [32]. Liver cysts affect about 75 % of ADPKD patients in their sixties [33] and usually gain in size and number as they do in the kidney. Women, especially those who have used hormones and/or have had multiple pregnancies, are more often and more severely affected, suggesting that estrogen influences hepatic cyst growth. Estrogen is known to stimulate the proliferation of cholangiocytes, and estrogen receptors are expressed in epithelial cells lining hepatic cysts. Cysts in other epithelial organs (e.g., seminal vesicles, pancreas, arachnoid membrane) and diverticulosis are also common. Among the cardiovascular co-morbidities, intracerebral aneurysms play a significant role and are present in about 8 % of ADPKD patients. The prevalence is even higher in patients with a positive family history for intracerebral aneurysms and/or subarachnoid hemorrhage. Cardiac valve disease, particularly mitral valve prolapse, can be diagnosed in approximately 25 % of patients with ADPKD.

## “Neoplasia in disguise”: cystogenesis shows similarities to tumorigenesis, but patients usually do not develop carcinoma

Although benign, the cystogenic process in PKD created by the sustained proliferation of epithelial cells resembles profiles often observed in tumors. Thus, some call ADPKD “neoplasia in disguise” [34, 35]. Many processes defective in ciliopathies, such as epithelial and planar cell polarity, play crucial roles in tumorigenesis [36]. Cilia are directly involved in cell cycle regulation and the coordination of cancer-related signaling molecules [37, 38]. Fortunately, however, renal cell carcinoma or any other tumor only rarely develops in PKD despite the frequency of hyperplastic polyps and microscopic adenomas in which aneuploid karyotypes with chromosomal extra-copies can often be determined. This phenomenon in cell growth and tumorigenesis is explained by the so-called aneuploidy paradox, which means that chromosome gains cause a proliferative disadvantage rather than advantage, as one may have assumed [39]. The integrity of cilia and the polycystin complex are required for the maintenance of mitotic spindle orientation and centrosome amplification [40, 41]. Interventions targeted at cell cycle regulation may thus be another smart drug approach, as demonstrated by beneficial effects of the cyclin-dependent kinase inhibitor roscovitine on cystic kidney disease in two PKD mouse models [42].

ADPKD exhibits some similarities to hereditary cancer syndromes, such as von Hippel-Lindau (VHL) syndrome and tuberous sclerosis (TSC). VHL is an autosomal dominant condition with a prevalence of about 1:30,000–40,000 caused by inactivating mutations of the *VHL* tumor suppressor gene and characterized by the development of hemangioblastomas in the brain, spinal cord, and retina often combined with renal clear cell carcinoma and pheochromocytoma [43]. Patients with VHL are also at increased risk for epididymal cystadenomas and cysts in the kidney and pancreas.

TSC is caused by an autosomal dominant germline mutation in either *TSC1* or *TSC2* and can affect a broad range of organs with a birth incidence of approximately 1:6,000 [44]. About 90 % of patients experience often intractable seizures and approximately one in two patients shows cognitive impairment, autism, or other behavioral disorders. Renal manifestations are the leading cause of death in adult patients with TSC [45]; cystic kidney disease occurs in 50 % of TSC patients, and angiomyolipomas are diagnosed in 80 %. Many other parts of the body (e.g., heart and brain) can also be affected by primarily non-cancerous tumors. A third of all female patients display pulmonary involvement, specifically lymphangioleiomyomatosis. Frequent skin manifestations in TSC are white spots, facial angiofibromas, and peri-/subungual fibromas.

Both TSC and VHL intersect with the primary cilium and the mTOR signaling network known to be upregulated in PKD and several tumor types [46]. The activity of mTOR is downregulated by polycystin-1 and the TSC1/TSC2 tumor suppressor complex, ultimately resulting in G1-cell cycle arrest and apoptosis. A synergistic role of polycystin-1 and the *TSC2* gene product tuberin has been suggested in the more severe and earlier onset PKD phenotype in individuals harboring a deletion encompassing the adjacent *TSC2* and *PKD1* genes on chromosome 16p13 compared to those with a *PKD1* mutation alone. A molecular explanation might be the fact that both proteins interact and tuberin traffics polycystin-1 to the plasma membrane [47].

## PKD phenocopies

### Hepatocyte nuclear factor 1ß-related disease

**Fig. 6:**
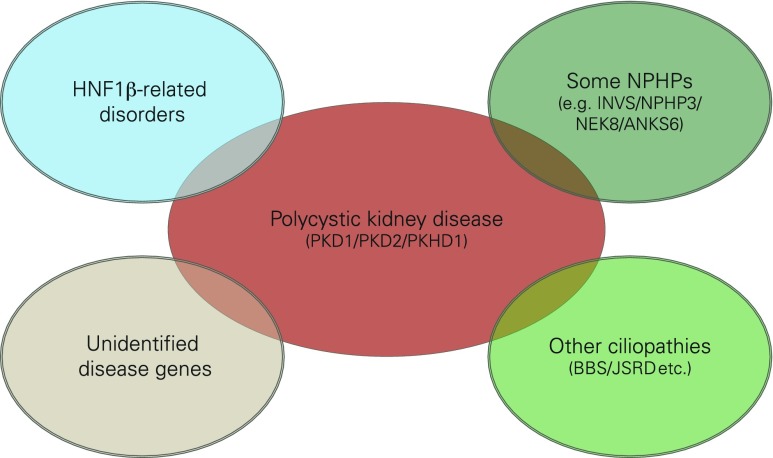
Several phenocopies of polycystic kidney disease (PKD) are known and mutations in the transcription factor hepatocyte nuclear factor 1 beta (*HNF1ß*) or genes that typically cause other ciliopathies, such as nephronophthisis (*NPHP*), Bardet-Biedl (*BBS*), and Joubert syndrome and related disorders (*JSRD*), can mimic PKD in some cases

Autosomal recessive PKD and ADPKD can be mimicked by mutations in a couple of other genes (Fig. 6). The transcription factor hepatocyte nuclear factor 1ß (HNF1ß) (*HNF1ß/TCF2* gene) plays a critical role in the diagnostics of cystic kidney disease. In line with a broad expression pattern from early embryonic development onwards, mutations in this transcription factor can result in a multitude of various phenotypes summarized as renal cyst and diabetes syndrome.

*HNF1ß* mutations follow an autosomal dominant mode of inheritance. However, as true for many transcription factors, incomplete penetrance, considerable variable expressivity, and a high fraction of spontaneous mutations have to be kept in mind when the family history is evaluated. Spontaneous de novo mutations are found in about 30–50 % of all HNF1ß patients. Almost half of all *HNF1ß* mutations are large deletions that usually encompass about 1.4 Mb of genomic DNA on 17q12, including *HNF1ß* and 14 other genes. In contrast to the deletion, the reciprocal duplication due to non-allelic homologous recombination in 17q12 is rare. Surprisingly, most patients carrying the microdeletion do not show a more severe phenotype than patients harboring an *HNF1ß* point mutation. However, there is now convincing evidence that a few of those patients with a large genomic rearrangement in 17q12 may have cognitive impairment, seizures, and other neurodevelopmental disorders (e.g., schizophrenia and autism spectrum disorders) [48, 49].

**Fig. 7:**
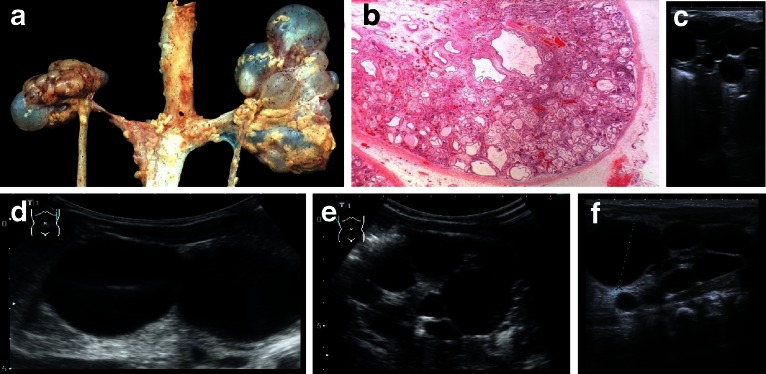
**a** Cystic kidney dysplasia due to early embryonic maldevelopment (Potter type II). Both kidneys have lost their reniform structure. The histologic (**b**) and ultrasonographic (**c**–**f**) pattern is irregular, with excessive connective tissue in between cysts of various sizes and at different locations

The most common renal phenotype that *HNF1ß* mutations lead to is cystic kidney dysplasia due to early embryonic maldevelopment (Potter type II—more often type IIa with enlarged kidneys than type IIb with renal hypodysplasia). In cystic dysplasia, both kidneys usually lose their reniform character (Fig. 7). The ultrasonographic pattern is irregular with excessive connective tissue in between various sizes and locations of cysts. Other features besides cystic kidneys and maturity-onset diabetes that are quite common in HNF1ß patients are genital tract abnormalities, hyperuricemia, increased liver enzymes, hypomagnesemia, and pancreatic atrophy with endocrine and exocrine insufficiency.

Clinical variability associated with *HNF1ß* mutations is extensive and not well understood to date. Phenotypes may range from prenatal manifestations to very mild symptoms in elderly family members. Renal phenotypes may even vary in the same patient, with cystic dysplasia (later renal "agenesis") in one kidney and a polycystic disease pattern in the other kidney (own unpublished data). Overall, *HNF1ß* mutations are thought to constitute the main cause of prenatally diagnosed bilateral hyperechogenic kidneys [50]. While many of these fetuses display normal-sized kidneys often with bilateral cortical cysts and normal amniotic fluid volume, HNF1ß patients may also show Potter’s sequence with oligo-/anhydramnios and massively enlarged polycystic kidneys (> +3 standard deviations) that mimic ARPKD. In accordance with these observations, mice with an *Hnf1ß* mutation were found to show a PKD-like phenotype with diminished *Pkhd1* expression [51]. HNF1ß is a master regulator with a major effect on many cystic kidney disease genes, such as *PKHD1*,* PKD2*, and *UMOD*, and there is evidence for a transcriptional network in PKD which is a plausible explanation of why some patients may resemble ARPKD or ADPKD patients.

There is also some evidence in the literature suggesting that HNF1ß may serve as a tumor suppressor. After identifying a 50-year-old patient with ovarian and chromophobe renal cell carcinoma, Rebouissou et al. found biallelic HNF1ß inactivation (by the combination of an *HNF1ß* germline mutation and a somatic gene deletion) in two of 12 chromophobe renal cell carcinomas [52]. Notably, the closely related homeodomain-containing transcription factor HNF1α, to which HNF1ß binds DNA as homo-dimers or hetero-dimers to activate transcription of targeted genes, exhibits a well-established tumor suppressor function in hepatocarcinogenesis and may also participate in the development of other tumors, such as colorectal and clear cell renal carcinomas. Recent data also suggest a broader role and association of HNF1ß with other tumors, such as ovarian and prostate cancer [53–55]. While it would be clearly overpowered to stoke fears about significant cancer risks in children carrying an *HNF1ß* germline mutation, it might be reasonable to provide adult mutation carriers with this information and discuss which standard investigations would be appropriate in each individual case (due to the absence of any official disease surveillance program).

### Certain types of nephronophthisis

**Fig. 8:**
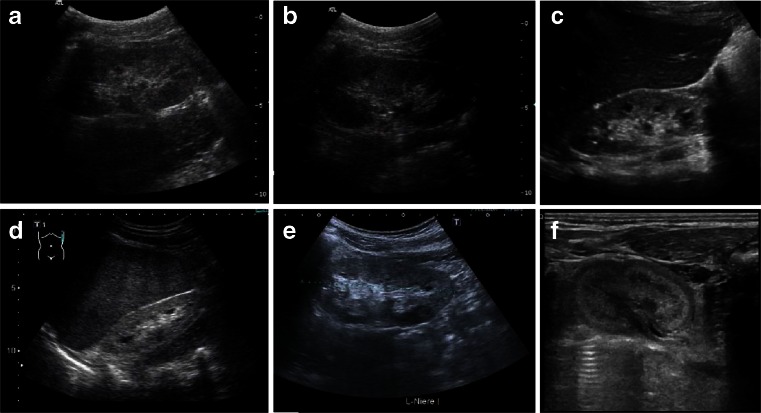
**a**–**f** Sonographic appearance of patients of different ages with nephronophthisis (NPHP). All patients show a typical NPHP pattern with normal or small-sized kidneys, enhanced echogenicity of the renal cortex, and reduced cortico-medullary differentiation (progressive with renal failure); cysts (e.g., patient in **c**) are usually a late sign and occur secondarily after patients have progressed to end-stage renal disease and are typically located at the cortico-medullary junction. **a**, **b** 10-year-old boy with homozygous *NPHP1* deletion, serum creatinine of 3 mg/dl, glomerular filtration rate of 20 ml/min and mild arterial hypertension. **c**, **d** 10- and 15-year-old patients with progressed clinical course. **e** 14-year-old boy with Senior–Loken syndrome carrying a homozygous deletion of the *NPHP1* gene. Peritoneal dialysis was started recently. **f** 2-day-old male patient with sonographic evidence of NPHP, polyuria, and systemic hypertension

**Fig. 9:**
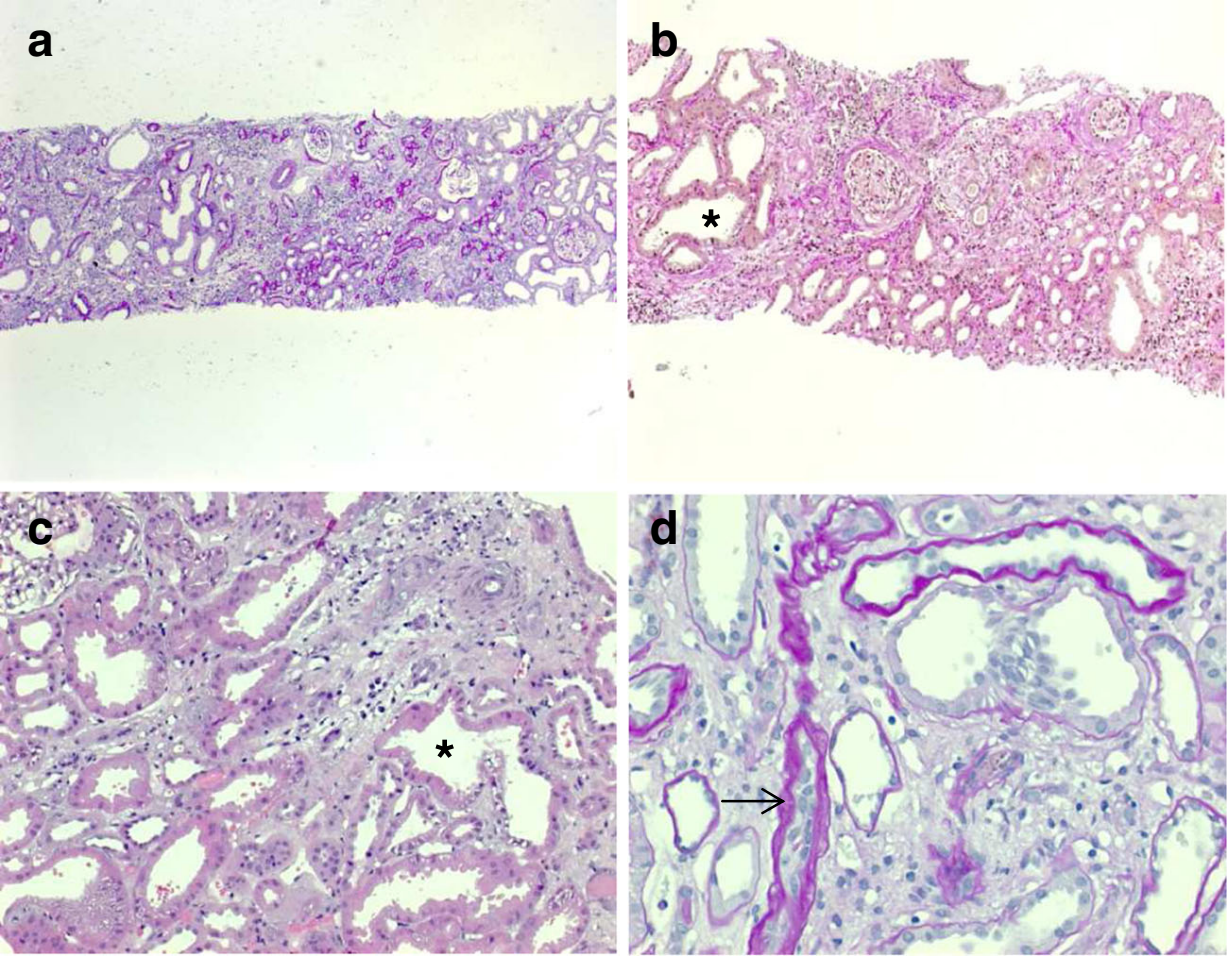
**a**–**d** Renal histology of a 14-year-old female with juvenile NPHP carrying a homozygous *NPHP1* deletion. Chronic tubulo-interstitial alterations with tubular basement membrane disintegration and thickening, focal tubular atrophy (*arrow* in **d**), cystic dilated tubuli (*asterisk* in **b** and **c**), and disproportionate tubulo-interstitial fibrosis with some inflammatory cells were observed

Nephronophthisis (NPHP) comprises a clinically and genetically heterogeneous group of autosomal recessive tubulointerstitial cystic kidney disorders. Characterization of NPHP proteins (nephrocystins) has considerably supported the understanding of processes involved in cystogenesis and cilia-related disorders. NPHP represents the most frequent genetic cause of ESRD in children and young adults and typically initially presents with a urinary concentrating defect [56, 57]. The most common form, juvenile NPHP, usually starts during the first decade of life with manifestations of polyuria, polydipsia, and anemia. Patients usually do not show arterial hypertension before the onset of renal failure. The ultrasound scans of NPHP patients generally show normal or small-sized kidneys with increased echogenicity and often also the loss of corticomedullary differentiation (Fig. 8). Cysts usually only occur as secondary manifestations after patients have progressed to end-stage renal failure and are typically located at the corticomedullary junction. Histologically, tubular basement membrane disintegration and thickening, tubular atrophy, and disproportionate tubulointerstitial fibrosis with minimal inflammation are observed [58] (Fig. 9).

Medullary cystic kidney disease (MCKD) is often regarded as the autosomal dominant counterpart of NPHP (NPHP–MCKD complex) with a usually later onset of renal failure than the recessive forms. The ciliary protein uromodulin (= Tamm–Horsfall glycoprotein), the most abundant protein in the urine of healthy individuals, plays a major role and is encoded by *UMOD* on chromosome 16p12 [59]. Mutations in this gene can lead to different tubulointerstitial nephropathies, including MCKD2, glomerulocystic kidney disease, and familial juvenile hyperuricemic nephropathy, which may also be caused by mutations in *TCF2* (HNF1ß) and *REN* (renin). Only recently has *MUC1*, a locus mapped more than a decade ago on chromosome 1q22, been identified as the gene underlying MCKD1 [60].

To date, about 20 genes have been described for recessive NPHP, plus *XPNPEP3* in which mutations cause an NPHP-like phenotype (*NPHP1L*). Given that these genes only account for about one-half of all NPHP cases, further heterogeneity can be expected. *NPHP1* on chromosome 2q13 is the most commonly mutated gene in NPHP. In large series of patients, a homozygous deletion of *NPHP1* was present in 20–40 % of cases [56, 57]; heterozygous deletions were found in another 6 % of patients, harboring a concomitant point mutation on the other parental *NPHP1* allele [57]. Other *NPHP* genes described to date may only contribute a minor part to the total mutational load of typical NPHP.

It is important to note that many *NPHP* genes are pleiotropic and can cause a much broader phenotypic spectrum (with other ciliopathies such as Senior–Loken, Joubert, Meckel, Ivemark, and Jeune syndrome) than just isolated NPHP. Mutations in a subset of *NPHP* genes can resemble PKD with enlarged kidneys and sometimes even prenatal manifestation of Potter’s sequence. Obviously, these respective NPHP members engage in a closely assembled protein–protein interaction sub-network (Fig. 1). We recently identified ANKS6 as an additional member and the central component of this novel cystoprotein module that connects NEK8 (NPHP9) to INVS (NPHP2) and NPHP3 [61]. Some preliminary evidence connects this module to oxygen-dependent hydroxylation and mitochondrial function.

### Other ciliopathies

In rare instances, PKD may be mimicked by mutations in genes that typically cause other, usually complex ciliopathies, such as Bardet–Biedl, Joubert and Meckel syndrome.

Bardet–Biedl syndrome (BBS) is characterized by obesity, hypogonadism, retinal degeneration, polydactyly, mental retardation, and renal malformations. Various additional features, such as hearing loss, diabetes mellitus, and other metabolic defects, have also been described. Renal disease is a major cause of morbidity and mortality in BBS and can be very heterogeneous in phenotype. While some kidneys manifest as NPHP with tubulointerstitial disease, others may exhibit findings resembling those usually seen in ARPKD and ADPKD patients, with enlarged, hyperechogenic kidneys and loss of cortico-medullary differentiation, with or without macrocysts. Currently, a total of 18 *BBS* genes and *ALMS1* as the gene for the closely overlapping Alstrom syndrome have been identified.

Meckel syndrome (MKS) is allelic to Joubert syndrome (JS/JBTS) and related disorders (JSRD) and is positioned at the severe end of the broad phenotypic spectrum of ciliopathies [62]. These multisystemic disorders are of an early developmental rather than degenerative nature. Clinically and genetically they are heterogeneous with more than 20 genes known to date. Classic disease manifestations in patients with MKS comprise occipital meningoencephalocele, cystic kidney dysplasia, hepatobiliary DPM, and postaxial polydactyly. Several other features, such as shortening and bowing of long tubular bones, heart defects, microphthalmia, and cleft lip/palate, have been reported. Survival beyond birth or the neonatal period is unusual, with the vast majority of babies dying in utero. Prognosis in patients with JS/JBTS varies considerably and largely depends on the extent and severity of organ involvement. The clinical hallmark is the so-called “molar tooth sign”, a complex mid-hindbrain malformation with cerebellar vermis hypoplasia visible on magnetic resonance imaging. The clinical correlates are developmental delay/mental retardation, neonatal irregular breathing, hypotonia, ataxia, and eye movement abnormalities [63]. Non-neurological features include polydactyly, retinal dystrophy, hepatic fibrosis, NPHP, and other cystic kidney disease phenotypes. In neonates and young infants, particular attention should be paid to respiratory and feeding problems.

## Why is it important to know the underlying genetic defect?

Although the clinical management of cystic kidney diseases of different origin is practically the same (until effective drugs are established), it is of major interest to know about the underlying genetic defect for the different reasons that are outlined below.

An often underestimated issue is the impact that a genetic diagnosis may have on many patients and their parents, i.e. the relevance of finally being able to give the disease a name.

Information on the genotype is also clearly of major importance when selecting patients for clinical trials and for future treatments. For example, patients carrying hypomorphic *PKD1/PKD2* alleles can be expected to progress slowly and may never develop ESRD; as such, the benefits may not outweigh the risks of any new treatment.

### Knowledge of genotype improves clinical management

It is always good to know your “enemy” in detail and what you are dealing with. Clinicians may find it helpful to have a better understanding of the specific condition they are treating and if there is any substantial risk for extrarenal disease manifestation that might benefit from early detection and disease monitoring ideally embedded in a multidisciplinary approach.

For example, despite all similarities, ARPKD and ADPKD are different diseases with different complications—and the clinician should take this into account. While in children with ARPKD portal hypertension is an invariable threat, ADPKD patients are only very rarely affected by DPM and congenital hepatic fibrosis. In contrast to ARPKD, cardiovascular co-morbidities, in particular intracerebral aneurysms (ICAs), play a significant role in ADPKD. An overall prevalence of asymptomatic ICAs of about 8 % has been estimated for ADPKD patients, with prevalence increasing with age, being about 23 % among the 60- to 69-year-old ADPKD patients in one study [64]. General recommendations do not exist, and the question of screening for ICAs in patients known to be affected with ADPKD is still a matter of debate and challenging in each individual case. Recent data support very selective screening for unruptured ICAs; widespread screening is definitely not indicated. Nevertheless, for both the patient and treating physician, it might be good to know about the increased risk in ADPKD, especially in patients with a positive family history who have a doubled relative risk [64, 65]. Fortunately, among children and adolescents, aneurysm rupture is very uncommon. Thus, screening might only be considered at the age of 20 years, especially when there is a positive family history.

Mutations in *NPHP* genes and other members of the ciliopathy spectrum may often lead to retinitis pigmentosa. Although there is currently no cure for this condition, an early diagnosis might help families cope better with the ultimate progression of the disease and choose the right course. It is widely accepted that the early learning of Braille correlates strongly with both academic and employment success later in life. Conversely, parents and patients can be reassured that visual impairment is not part of the disease spectrum if, for example, mutations in HNF1ß or any of the PKD genes have been identified.

Another good example is Alstrom syndrome which overlaps with BBS and typically shows retinal dystrophy, obesity, and endocrinological features. However, in contrast to BBS, most patients with Alstrom syndrome have normal intelligence and do not show polydactyly; they usually develop more severe sensorineural deafness and early-onset type 2 diabetes mellitus. Other features important for proper clinical surveillance are progressive pulmonary, hepatic and renal failure, and different cardiac manifestations. More than half of all Alstrom patients develop cardiac failure as a result of dilated or restrictive cardiomyopathy.

In some cases, a genetic diagnosis ends an often lengthy odyssey for the patients and families or may help to avoid invasive procedures (e.g., kidney or liver biopsy).

### Genetic counseling

It is only possible to discuss various aspects of diseases, such as clinical course, disease spectrum, and recurrence risk, in greater detail if the nature of the patient’s genotype is known.

Understandably, for parents who already have an affected child and who wish to have more children, knowing whether they carry a 50 % (as for autosomal dominantly inherited disorders), 25 % (as for recessive diseases), or practically no risk of recurrence has a huge effect on their decisions. The term “practically no risk of recurrence” holds true when the mutation in the patient arose de novo. A 100 % guarantee cannot be given because of theoretically possible germline mosaicism in one of the parents; thus, the use of “practically no recurrence risk”. Autosomal dominant de novo mutations are present in about 30–50 % of all HNF1ß patients. These may also occur, in other dominant disorders, such as ADPKD, but usually with a much less frequency (in up to 5 % of ADPKD patients). The patients themselves harboring such a de novo mutation carry a 50 % recurrence risk for their own children; however, for parents and their families the elucidation of a de novo event in the pedigree’s index patient is a big relief.

A clear genetic diagnosis is also of interest to the rest of the family in X-linked disorders. A frequent scenario for genetic counseling is the healthy sister of an affected patient who is afraid that her brother bears an X-linked mutation and that she might be a carrier with a substantial disease risk for her own children (then 50 % for boys).

With improved clinical management even patients with early disease manifestation often reach adulthood and may wish to have their own children. For these patients, it is of major importance to know if they are afflicted by a recessive or a dominant disease. While a dominant disease type will mean a 50 % recurrence risk, recessive inheritance usually results in a <1 % risk for offspring unless consanguineous marriage takes place, the partner is equally affected, or a patient of the same disease is present in the partner’s pedigree. In particular, for male patients it might also be of interest to distinguish between autosomal and X-linked inheritance, as a father affected by an X-linked disorder can never have affected sons.

### Prenatal diagnosis

Many parents of children with ARPKD and other cystic kidney diseases seek early and reliable prenatal diagnosis to guide future family planning. The main reasons cited for prenatal studies are the usually significant risk of recurrence, frequently devastating early manifestations of the disease, and often comparable clinical courses among affected siblings.

An early and reliable prenatal diagnosis in “at risk” families is usually only feasible by molecular genetic analysis. For ARPKD, the dilemma with non-invasive prenatal imaging techniques is that patients are typically identified by ultrasound only late in pregnancy or even only at birth. Even with state-of-the-art technology, fetal sonography at the time when termination of pregnancy is usually performed frequently fails to detect enlargement and increased echogenicity of kidneys or oligohydramnios. In the past, indirect haplotype-based linkage analysis has often been performed for ARPKD in terms of prenatal diagnosis. However, due to the aforementioned reasons with phenocopies and evidence for further heterogeneity in ARPKD, haplotype-based prenatal diagnostics is no longer state of the art and regarded as too risky without knowledge of *PKHD1* mutational status. It should only be performed in those families in which the diagnosis has been previously proven unequivocally. Still, molecular prenatal diagnostics is usually done after chorionic villus sampling, an invasive procedure undertaken quite late in the pregnancy (no earlier than the 10–12th week of gestation). Interested families should also be informed of the possibility of pre-implantation genetic diagnosis at some single diagnostic centers and that this procedure always requires a high degree of coordination and work-up in advance. A future scenario might be non-invasive prenatal testing of the fetal genotype by screening a maternal blood sample.

## Conclusions

We have learned during recent years that there is huge clinical and genetic variability in cystic and polycystic kidney disease even within the same family. Classical Mendelian inheritance patterns with single-locus allelism may sometimes appear to be insufficient to explain such phenomena as variable expressivity and incomplete penetrance. It is rather likely that stochastic, epigenetic, and environmental factors modify the phenotype. Furthermore, oligogenic inheritance with variations across multiple sites may further influence the clinical outcome in some individuals [2]. Such “additional” alleles, the so-called second-site modifiers, may exert an aggravating effect in an epistatic manner and contribute to early and severe disease expression in some patients. In these patients it is hypothesized that the reduced dosage of disease proteins (“dosage-sensitive network”) may disturb converging pathways, network integrity, and cell homeostasis, which may ultimately explain the more severe clinical course [66, 67]. Further research is on-going and will hopefully provide data on which firm conclusions can be drawn.

Next-generation sequencing (NGS) provides a rapidly growing insight in these challenging issues and has revolutionized genetic diagnostics. Conventional (Sanger) sequencing facilities are expensive with limited capacities and only allow for a stepwise approach dependent on mutation frequencies and the ethnic origin and phenotype of the patient. In the case of parental consanguinity and in multiplex pedigrees with more than one affected child, linkage analysis with subsequent gene sequencing in the case of compatible haplotypes might be an option. However, ultimately, all of these conventional approaches are usually more cost- and time-intensive than (targeted) NGS-based approaches that allow large-scale parallel sequencing of all disease genes that may have to be discussed in a patient. Given overlapping disease phenotypes and extensive allelism, usually multiple, often dozens, genes have to be considered to be disease-relevant. Notably, NGS is not NGS, and approaches are definitely not the same. More than most other in vitro diagnostic tests, NGS needs strict consideration of certain quality criteria. However, if all this is warranted, NGS does not only improve the throughput, but also the quality of genetic diagnostics. For example, in contrast to Sanger sequencing, by using NGS it is possible to detect mosaicism and copy-number variations. For most genes, large deletions/duplications make up about 5–10 % of the total mutational spectrum of the respective gene, and for some genes (e.g., in HNF1ß, almost 50 % of patients have large deletions) the proportion is even much higher. Overall, parallel analysis of all targeted disease genes allows for a better interpretation of identified variants and avoids genetic misdiagnoses. Such a targeted NGS-based approach considerably increases the detection rate, allowing the major disease gene to be clearly identified in the great majority of cases. In terms of diagnostics, specific NGS panels, which ideally should have a critical minimal size (i.e., comprising a sufficient number of targeted genes), are thought to be currently better than exome sequencing in the first place. However, families who are analyzed by an NGS panel, but for whom the mutations in known genes were not identified, should be offered exome sequencing, which is already provided on a diagnostic basis by some laboratories. Importantly, a much more detailed form of informed consent is required for exome sequencing.

Genetic technologies are evolving rapidly. Some people think that with these new opportunities a deep clinical knowledge becomes outdated. However, the opposite is the case, and a structured, critically reflected and multi-disciplinary approach becomes even more important than ever before. Interpretation of data is now the challenge, and bench and bedside clearly benefit from smart bioinformatic algorithms and support.
